# The Protein Expression of PDL1 Is Highly Correlated with Those of eIF2*α* and ATF4 in Lung Cancer

**DOI:** 10.1155/2018/5068701

**Published:** 2018-09-16

**Authors:** Liang-Che Chang, Tzu-Ping Chen, Wei-Ke Kuo, Chung-Ching Hua

**Affiliations:** ^1^Department of Pathology, Chang Gung Memorial Hospital, Keelung and Chang Gung University, Keelung, Taiwan; ^2^Division of Thoracic and Cardiovascular Surgery, Chang Gung Memorial Hospital, Keelung and Chang Gung University, Keelung, Taiwan; ^3^Division of Pulmonary, Critical Care and Sleep Medicine, Chang Gung Memorial Hospital, Keelung and Chang Gung University, Keelung, Taiwan

## Abstract

**Introduction:**

The expression of programmed death 1 (PD1) and programmed death ligand 1 (PDL1) can be induced by the interferon (IFN)/signal transducer and activator of transcription (STAT) pathway. The PD1/PDL1 reverse signaling can activate the eukaryotic translation initiation factor 2 (eIF2*α*)/activating transcription factor 4 (ATF4) pathway which in turn regulates the expression of IFN regulatory factor (IRF) 7 and IFN*α*. The eIF2*α*/ATF4 pathway is responsible for the integrated stress response (ISR) of unfolded protein response (UPR) which can affect immune cell function in tumor microenvironment.

**Materials and Methods:**

The protein levels of PDL1, IRF1, IRF7, STAT1, STAT2, IFNAR1, eIF2*α*, and ATF4 in the normal and tumor tissues of 27 subjects with lung cancer were determined by Western blot.

**Results:**

The protein level of PDL1 was significantly correlated with those of IRF1, eIF2*α*, and ATF4 in the tissues of all subjects and the subgroup of squamous cell carcinoma but not in the normal tissue of adenocarcinoma. The protein levels of IRF1, eIF2*α*, and ATF4 were consistently correlated in the tumor tissues but to various extents in the normal ones. The protein level of PDL1 was not correlated with those of STAT1 and STAT2 in all the tissues.

**Conclusion:**

The PDL1 expression in lung cancer may be independent of STAT1 and STAT2. The PD1/PDL1 axis and UPR/ISR may be closely associated in the tumor tissues of lung cancer.

## 1. Introduction

Immune checkpoint inhibitors (ICI) like anti-PD1 (programmed death 1) or anti-PDL1 (programmed death ligand 1) antibodies are effective in treating various neoplasms, including lung cancer [1]. The first-line use of pembrolizumab, an anti-PD1 antibody, has longer progression-free and overall survival than platinum-based chemotherapy in advanced non-small-cell lung cancer (NSCLC) with PDL1 expression at least 50% of tumor cells [2]. Similar findings have been observed in the second line of ICI in advanced NSCLC [3–5]. PDL1 tumor overexpression is associated with high response to anti-PD1 antibody in pretreated NSCLC [6]. The expression of PDL1 in lung cancer is important in selecting patient to treat with ICI.

**Figure 1:**
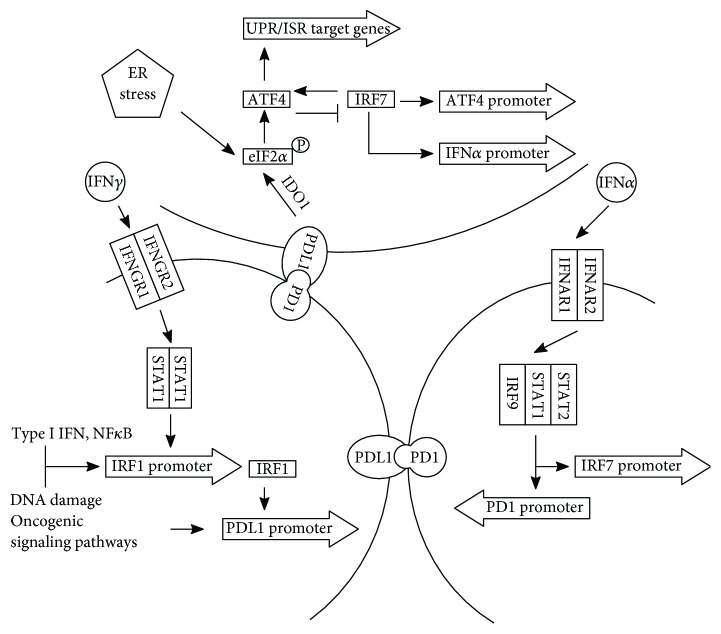
PDL1 expression can be upregulated by oncogenic signaling or the IFN*γ*/STAT1/IRF1 pathways. IRF1 expression can be upregulated by type I and type II IFN, NF-*κ*B, and DNA damage. The binding of IFN*α* to IFNAR1/IFNAR1 leads to the formation of ISGF3 (a heterotrimer of STAT1, STAT2, and IRF9) which can upregulate the expression of PD1 and IRF7. The binding of PD1 to PDL1 can have reverse signaling that upregulates IDO1 which can cause phosphorylation of eIF2*α*. The phosphorylated eIF2*α* can activate ATF4 which can upregulate the expression of UPR/ISR target genes and inhibit the activity of IRF7. IRF7 can upregulate the expression of ATF4 and IFN*α*. ATF4, activating transcription factor 4; eIF2*α*, eukaryotic translation initiation factor 2; IDO1, indoleamine 2,3-dioxygenase 1; IFN, interferon; IFNAR, interferon alpha/beta receptor; IRF, interferon regulatory factor; ISGF3, interferon-stimulated gene factor 3; PD1, programmed death 1; PDL1, programmed death ligand 1; STAT, signal transducer and activator of transcription.

The proliferation and effector functions of T cell can be inhibited via the binding of PDL1 or PDL2 to PD1 on its surface [7]. PDL1 is expressed on many immune cells and nonimmune cells and is upregulated by proinflammatory cytokines such as interferon (IFN) *γ* and IL4 through signal transducer and activator of transcription 1 (STAT1) and IFN regulatory factor 1 (IRF1) [7, 8]. Tumor cells can induce the expression of PDL1 directly through the constitutive oncogenic pathways or indirectly with the help of T cell and the activation of IFN/STAT pathway [9]. Inflammatory cells, including activated T cells, contribute to the progression of malignancies [10]. In chronically activated T cells, IFN*α* causes prolonged PD1 transcription through the binding of activated IFN-stimulated gene factor 3 (ISGF3), a heterotrimer of STAT1 and STAT2 in association with IRF9 [11], to the PD1 promoter [12]. The PD1/PDL1 axis is regulated by the IFN/STAT pathway (Figure 1).

Tumor mutational burden (TMB) and consequent neoantigen generation are crucial to tumor cell recognition by the immune system [13]. TMB is an independent predictor of response to ICI [14]. Tumor mutations can also induce unfolded protein response (UPR) through neoantigen-independent mechanisms [15]. UPR signaling components may induce T cell exhaustion and plays a role in the development of tumor resistance to ICI [16]. UPR, induced by endoplasmic reticulum (ER) stress, and other non-ER stresses can converge on eukaryotic translation initiation factor 2 (eIF2*α*) phosphorylation which enhance the transcription of UPR/integrated stress response (ISR) target genes through the activation of activating transcription factor 4 (ATF4) [17]. IRF7 upregulates ATF4 activity and expression, whereas ATF4 in return inhibits IRF7 activation [18]. IRF7 forms a positive feedback loop with IFN*α* [19]. INF*α* can bind the heterodimer of IFN alpha/beta receptor 1 (IFNAR1)/IFNAR2 and activate STAT-dependent pathways with or without the participation of STAT1 [20]. The eIF2*α*/ATF4/IRF7 pathway may interact with the PD1/PDL1 axis through signaling transduction of the IFN*α*/IFNAR/STAT pathway (Figure 1).

PDL1 immunohistochemical (IHC) stain has been the predictive biomarker for the treatment response of ICI like nivolumab and pembrolizumab [3–5]. However, 0% to 17% of patients with “negative” PDL1 IHC stain will still respond to anti-PD1 therapy, and patients with higher TBM have better responses to ICI [21]. Melanoma with negative PDL1 expression by IHC has a low nonsynonymous mutation rate [22]. TBM can induce UPR [15] which may affect the PD1/PDL1 axis through the eIF2*α*/ATF4 pathway [12, 17–20]. This study used Western blot to investigate the correlations between the protein expression of PDL1 and those of the IFN/STAT and the UPR/ISR pathways in lung cancer tissues.

## 2. Materials and Methods

Lung cancer subjects after surgery were selected for inclusion retrospectively from the biobank database of Chang Gung Memorial Hospital, Keelung. The frozen tumor and normal lung tissues of 27 lung cancer subjects with their deidentified clinicopathologic features were obtained from the biobank. The work was approved by the Institutional Review Board of Chang Gung Memorial Hospital (no. 106-1789C).

### 2.1. Protein Extraction

The tissues frozen in liquid nitrogen were thawed at room temperature and washed by 1x Dulbecco's phosphate-buffered saline. The washed tissues with 500 *μ*l 1x cell lysis buffer in 2 ml reinforced homogenization tubes prefilled with 2.8 mm zirconium oxide beads (CK28-R, Bertin Technologies, Montigny-le Bretonneux, France) were ground by using a Precellys homogeniser (Bertin Technologies) at 6000 rpm for 2 courses of 10 sec × 3 times with an interval of 5 minutes. The ground tissue was added with 10 *μ*l of each phosphatase and protease inhibitor cocktail and was left on the ice for 10 minutes after vortex mixing. After centrifugation with 14,000 g at 4°C for 15 minutes, the supernatant was collected, quantitated, and stored at −80°C for the later gel electrophoresis.

### 2.2. Western Blot

**Figure 2:**
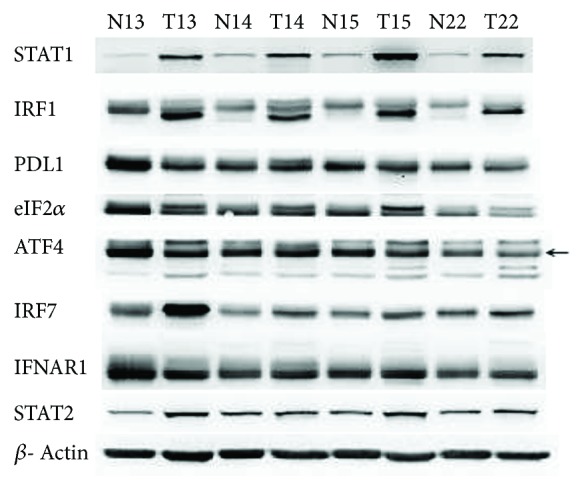
Examples of protein electrophoresis. T: tumor tissue, N: normal tissue.

The primary antibodies used were anti-STAT1 (ab3987; Abcam, Cambridge, MA; 1 : 1000), anti-IRF1 (ab186384; Abcam; 1 : 1000), anti-PDL1 (ab205921; Abcam; 1 : 5000), anti-eIF2*α* (ab169521; Abcam; 1 : 2500), anti-ATF4 (ab184909; Abcam; 1 : 1000), anti-IRF7 (ab109255; Abcam; 1 : 1000), anti-IFNAR1 (ab180812; Abcam; 1 : 1000), and anti-STAT2 (ab106094; Abcam; 1 : 1000). The secondary antibodies were obtained from Santa Cruz (sc-2005) and Abcam (ab-6721). The proteins were separated on 12% Tris-glycine PAGE gels (Acryl/Bis 29:1, Amresco #0311, Solon, Ohio, USA) at 110 V for 2 hours and were then transferred to a PVDF membrane (Immobilon-P membranes, Millipore #IPVH00010, Billerica, MA, USA) at 400 mA for 2.5 hours. The membrane was blocked with 5% skim milk in phosphate-buffered saline containing 0.1% Tween-20 (PBST) at room temperature for 1 hour and then incubated with primary antibodies at 4°C overnight. After the membrane was washed 4 times with PBST for 5 minutes each time, the secondary antibody was added and the membrane was incubated for 1 hour at room temperature. After being washed 4 times with PBST for 5 minutes each time, the chemiluminescent detection was performed using Luminata Forte Western HRP Substrate (Millipore, #WBLUF 0500) and visualized using the Bio-Rad VersaDoc400 imaging system. The expression of each protein was normalized to that of *β*-actin. An example of protein electrophoresis is illustrated in Figure 2.

## 3. Statistical Analysis

Paired or unpaired Wilcoxon signed-rank test was used to detect the differences of protein expressions between tissues. The correlations between protein expressions were assessed by Spearman's rho test. Hierarchical clustering with nonparametric multiscale bootstrap resampling was performed using the R statistical package (http://www.r-project.org/) [23] with the pvclust tool (http://www.is.titech.ac.jp/Bshimo/prog/pvclust/) [24], with correlations between the variables or clusters presented by the approximately unbiased (au) probability value. A cluster with an au value > 0.95 rejects the hypothesis of “the cluster does not exist” with a significance level of 0.05. The following options in pvclust were used: method: hclust = “average”; nboot = 10,000; *r* = seq (0.5, 1.4, by = 0.1). A *P* value < 0.05 was considered statistically significant.

## 4. Results

The pathologic features of 27 lung cancer subjects are shown in Table 1.

### 4.1. Differences between the Protein Expressions of Tissues

Protein expression of molecules in the normal and tumor tissues is listed in Table 2. The tumor tissue had higher expressions of IRF7 and STAT2 than the normal tissue in the all subject group (*p* value 0.003 and 0.01, respectively) and in the subgroup of squamous cell carcinoma (SCC) (*p* value 0.03 and 0.02, respectively). Adenocarcinoma had lower IRF7 levels in the tumor tissue (*p* value 0.001) and higher STAT2 expressions in the normal tissue (*p* value 0.001) than SCC. The normal tissue had lower IFNAR1 levels than the tumor tissue in the all subject group (*p* value 0.02) but not in the subgroup of adenocarcinoma or SCC. The protein levels of all molecules in both the normal and tumor tissues were not affected by nodal metastasis or tumor differentiation (*p* > 0.05).

### 4.2. Correlations between the Protein Expressions of Tissues

**Figure 3:**
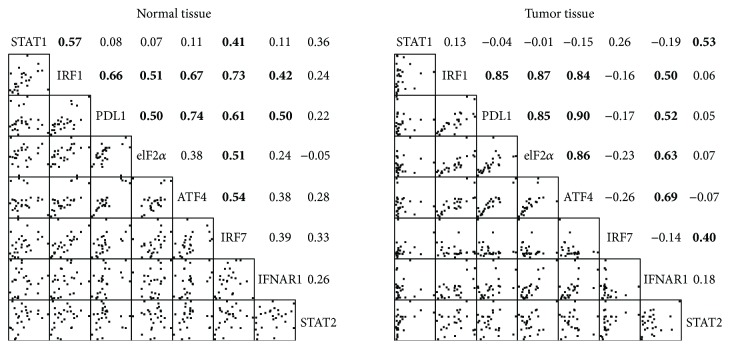
Spearman's correlation coefficients of protein levels between molecules in the normal or tumor tissue of all the lung cancer subjects. Bold number denotes a correlation with a *p* value < 0.05. Bivariate scatterplots are shown in the left lower half of each plot.

Figure 3 shows the Spearman's rho correlation coefficients between the protein levels in the normal and tumor tissues of the all subject group. The correlations among the protein levels of IRF1, PDL1, eIF2*α*, and ATF4 were significant in the tumor tissues, and similar patterns were found in the normal tissues except those between eIF2*α* and ATF4. The protein levels of STAT1 and IRF1 were significantly correlated in the normal but not in the tumor tissues. The protein expression of IFNAR1 was significantly correlated with those of IRF1 and PDL1 in both tissues but with those of ATF4 and eIF2*α* only in the tumor tissue. The protein level of IRF7 was correlated only with that of STAT2 in the tumor tissue but with those of molecules other than STAT2 and IFNAR1 in the normal tissue.

**Figure 4:**
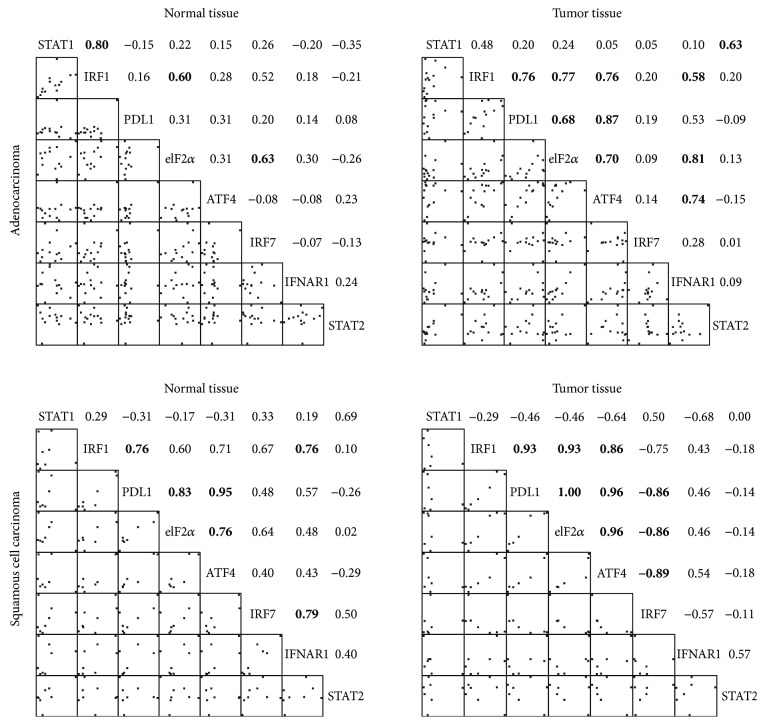
Spearman's correlation coefficients of protein levels between molecules in the normal or tumor tissue of subjects in the subgroups of adenocarcinoma and squamous cell carcinoma. Bold number denotes a correlation with a *p* value < 0.05. Bivariate scatterplots are shown in the left lower half of each plot.

The correlations between the protein levels in the subgroups of adenocarcinoma and SCC are shown in Figure 4. Significant correlations among the protein levels of PDL1, eIF2*α*, and ATF4 and between those of IRF1 and PDL1 were observed in the tumor tissues of both subgroups and in the normal tissue of SCC. The level of PDL1 was not correlated with those of STAT1 and STAT2 in all the tissues of subgroups. The level of IRF1 was correlated with that of STAT1 in the normal tissue of adenocarcinoma. The level of IRF7 was negatively correlated with those of PDL1, eIF2*α*, and ATF4 in the tumor tissue of SCC. The largest diameter or the size of tumor was not correlated with the protein levels of molecules.

### 4.3. Hierarchical Cluster Dendrogram

**Figure 5:**
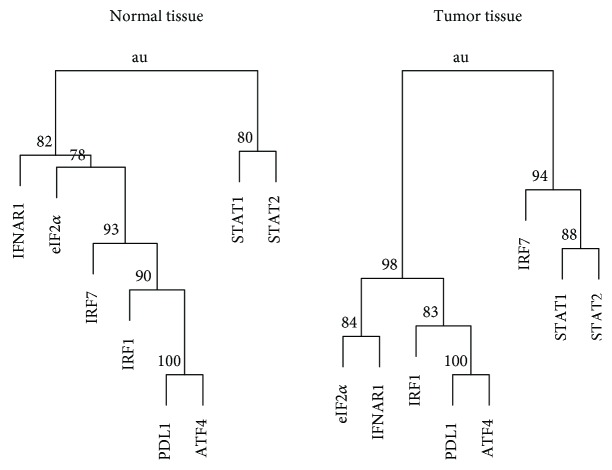
Cluster dendrograms showing the approximately unbiased probability (au) for the protein levels in the normal or tumor tissue of all the lung cancer subjects. An au value greater than 95% indicates that a cluster formed by the variables downstream of the node is strongly supported by data.

The hierarchical cluster dendrograms for the protein levels in the normal and tumor tissues of the all subject group are shown in Figure 5. The protein levels of ATF4 and PDL1 had the closest proximity in both tissues, and they formed a cluster with those of IRF1, eIF2*α*, and IFNAR1 in the tumor tissue.

## 5. Discussion

The protein levels of ATF4, eIF2*α*, and IRF1 were correlated with that of PDL1 in all the tissues except the normal one of adenocarcinoma. The protein levels of ATF4 and eIF2*α* were significantly correlated in the tumor tissues of all subjects and the subgroups of adenocarcinoma and SCC and in the normal one of SCC. The protein levels of IRF1, eIF2*α*, ATF4, and PDL1 in the tumor tissues of the all subject group formed a cluster in the hierarchical cluster dendrograms. The protein level of PDL1 was not correlated with those of STAT1 and STAT2 in all the tissues.

T cell tolerance induced by the PD1/PDL1 binding can be usurped by tumors to attenuate tumor immunity [25]. The PDL1 expression on tumor cells can be upregulated by intrinsic oncogenic signaling pathways or by surrounding T cells through the IFN*γ*/STAT1/IRF1 pathway and chronic type I IFN exposure [7–9, 11]. IFN*α* can induce the formation of ISGF3 complex which in turn binds the PD1 promoter and increase its transcription in chronically activated T cells [12, 26]. The PD1/PDL1 reverse signaling can induce the expression of indoleamine 2,3-dioxygenase 1 (IDO1) in the tumor cells, which in turn causes phosphorylation of eIF2*α* with subsequent activation of ATF4 [17] and inhibition of IRF7 activity [18] in Treg, dendritic cells, myeloid-derived suppressor cells, and endothelial cells [27]. IRF7 is essential for the induction of type I IFN genes [19]. Type I IFN might form a feedback loop with the PD1/PDL1 axis through the eIF2*α*/ATF4 pathway.

The protein levels of IRF1, eIF2*α*, ATF4, and PDL1 in the tumor tissues of the all subject group were significantly correlated with each other and formed a cluster in dendrogram (Figure 5). The results may suggest that the PD1/PDL1 reverse signaling may activate the eIF2*α*/ATF4 pathway in tumor surrounding cells [27] and IRF1 may be active in regulating PDL1 expression in the tumor tissue [7–9]. IRF1 is the key regulator of PDL1 promoter [28], and it is constitutively expressed and inducible by IFN and DNA damage [29]. Lung cancer patients have upregulated inducible nitric oxide (NO) synthase in tissue and increase exhaled NO level [30]. NO can induce both DNA damage and UPR [31], which is regulated in part by eIF2*α* and ATF4 of the ISR pathway [17]. The significant correlations between the protein levels of IRF1 and eIF2*α* or ATF4 in the tumor tissues may result from high ER stress which trigger UPR in cancer [32]. Tumor tissue may have high UPR due to the Warburg effect and the lack of efficient microvasculature as seen in normal tissue [32]. SCC had significant correlations between IRF1 and eIF2*α* or ATF4 in the tumor but not in the normal tissue. The results are in agreement with the presence of high ER stress in tumor [32]. The correlations between eIF2*α* and ATF4 were insignificant in the normal tissue of adenocarcinoma. ATF4 is efficiently translated only in stress conditions with its expression regulated by eIF2*α* phosphorylation, which can be induced by UPR that is active under high TMB [15, 17, 33]. TMB is lower in adenocarcinoma than SCC of lung, and normal tissues may have burdens of somatic mutations broadly similar to tumor of the same cell type [34]. The significant correlations among eIF2*α*, ATF4, IRF1, and PDL1 in lung cancer may result from ER stress-induced UPR/ISR and the PD1/PDL1 reverse signaling.

The protein expression of IRF7 in the tumor tissue of SCC was higher than those in the corresponding normal tissue and the tumor tissue of adenocarcinoma. IRF7 expression can be regulated by type I IFN and TNF*α* through STATs and NF-*κ*B, respectively [35, 36]. High TMB in the tumor tissue of lung SCC [34] may activate the eIF2*α*/ATF4 pathway via neoantigen-induced UPR [15], which can induce NF-*κ*B activation [31]. However, IRF7 promoter has been found to be hypermethylated in lung cancer [36, 37]. The high expression of IRF7 in the tumor tissue of SCC may come from the tumor surrounding cells or by other mechanism like posttranslational regulation [36]. IRF7 upregulates ATF4 expression, and ATF4 inhibits the transcription of IRF7 [18]. The expression of IRF7 was negatively correlated with those of eIF2*α* and ATF4 in the tumor tissue of SCC. IRF7 enhances the PDL1 expression by directly promoting its transcription [38]. However, the protein expression of PDL1 was negatively correlated with that of IRF7 in the tumor tissue of SCC. The ability of IRF7 to promote PDL1 transcription may be not as potent as IRF1, and the effect of PD1/PDL1 reverse signaling on eIF2*α*/ATF4 activation with subsequent inhibition of IRF7 expression may result in a negative correlation between the protein levels of IRF7 and PDL1 in the tumor tissue of SCC.

Although STAT1 and STAT2 activate IRF1 [7–9, 28], the protein level of IRF1 was not correlated with that of STAT2 in all the tissues and had a significant correlation with that of STAT1 only in the normal tissue of lung adenocarcinoma. IRF1 is the key regulator of PDL1 promoter [28], and its expression is inducible by type I/II IFN and cytokines like TNF*α* and NF-*κ*B [11, 39]. The protein level of PDL1 was not significantly correlated with those of STAT1 and STAT2 in all the tissues. The regulation of PDL1 expression in lung cancer may depend on factors other than STAT1 and STAT2 such as NF-*κ*B [7]. The expression of IFNAR1 was correlated with that of IRF1 in the tumor tissue of adenocarcinoma and those of IRF1 and IRF7 in the normal tissue of SCC. The binding of IFN*α*/*β* to IFNAR1/IFNAR2 can induce the transcription of IRF7 through ISGF3 and that of IRF1 by the signaling traduced by STAT homodimers or heterodimers and STAT/CRKL (v-crk sarcoma virus CT10 oncogene homolog (avian)-like) [40]. Although the expressions of IFNAR1, IRF1, and IRF7 had different patterns of correlations among tissues, they were not correlated with those of STAT1 and STAT2. The effects of IFN signaling pathway on the PD1/PDL1 axis may not depend on STAT1 or STAT2 in lung cancer.

## 6. Conclusions

Abnormal ER stress is a critical regulator of immune function in tumor microenvironment and is important in immunotherapy [32]. The eIF2*α*/ATF4 pathway can help the transformed cells to evade oncogene-induced senescence and oxidative stress [33]. The PD1/PDL1 reverse signaling can activate the eIF2*α*/ATF4 pathway through IDO1 [17, 27] whose inhibitors are actively undergoing clinical trials in combination with anti-PD1 or anti-PDL1 antibodies to treat cancers [41]. The different correlation profiles of IRF1, eIF2*α*, ATF4, and PDL1 protein expressions in the normal and tumor tissues of lung cancer, especially adenocarcinoma, may be helpful in selecting conditions for combining ICI and inhibitors against IDO1 [41] or modulators of ER stress. However, this study is retrospective and correlational in nature and has limitations of small sample size, heterogeneous population, and limited clinicopathological information. A large-scale prospective study with the exploration of molecular mechanisms is needed to verify the preliminary results found in this one.

## Figures and Tables

**Table 1:** Pathologic features.

Histologic type		
	Adenocarcinoma	14
	Squamous	8
	Adenosquamous	2
	Pleomorphic carcinoma	2
	Carcinoma	1
T		
	1	5
	2	16
	3	4
	4	2
N		
	0	16
	1	8
	2	3
Differentiation		
	Well & moderate	18
	Poor	9
Tumor size		
	Largest diameter, cm	4.2 ± 2.5
	Volume, cm^3^^∗^	48.1 ± 88.4

^∗^Only the largest diameter was recorded in 3 pathologic reports.

**Table 2:** Protein expressions of subjects with paired samples of both the tumor and normal tissues.

	All			Adenocarcinoma			Squamous cell carcinoma		
	*N* = 27		*p*	*N* = 14		*p*	*N* = 8		*p*
	Normal tissue	Tumor tissue		Normal tissue	Tumor tissue		Normal tissue	Tumor tissue	
STAT1	1.49 (0.68–3.46)	1.84 (0.55–21.8)	[0.06]	1.66 (0.8–3.02) {0.13}	1.95 (0.55–14.5) {0.53}	[0.502]	1.09 (0.63–3.46)	1.46 (0.83–5.77)	[0.69]
IRF1	2.23 (1.08–4.45)	2.32 (0.45–4.38)	[0.69]	2.25 (1.24–4.45) {0.21}	2.40 (0.56–4.38) {0.69}	[0.95]	1.69 (1.18–2.92)	1.69 (0.45–4.35)	[0.94]
PDL1	13.5 (5.87–32.9)	12.6 (2.27–36.4)	[0.26]	13.5 (9.85–32.9) {0.33}	13.5 (2.57–21.3) {0.91}	[0.30]	10.7 (7.11–29.9)	11.7 (2.27–36.4)	[0.94]
eIF2*α*	4.57 (0.55–7.67)	4.81 (1.21–14.7)	[0.40]	4.64 (2.17–6.51) {0.66}	4.99 (2.08–14.7) {0.53}	[0.39]	4.23 (2.67–7.67)	3.75 (1.21–8.11)	[1.00]
ATF4	2.43 (0.83–4.79)	2.10 (0.37–5.96)	[0.21]	2.50 (1.76–4.30) {0.13}	2.57 (0.56–3.16) {0.64}	[0.39]	1.79 (0.97–4.60)	1.63 (0.37–5.96)	[0.94]
INFAR1	0.12 (0.02–0.23)	0.13 (0.02–0.50)	[0.02]	0.12 (0.02–0.23) {0.61}	0.15 (0.02–0.50) {0.53}	[0.02]	0.09 (0.02–0.23)	0.15 (0.03–0.38)	[0.11]
IRF7	1.30 (0.65–2.26)	1.74 (0.17–12.8)	[0.003]	1.28 (0.90–2.56) {0.13}	1.43 (0.17–2.65) {0.001}	[0.58]	1.03 (0.66–1.94)	3.31 (1.65–12.83)	[0.03]
STAT2	6.70 (0.74–10.5)	8.54 (3.51–14.0)	[0.01]	7.36 (0.99–10.5) {0.001}	6.74 (3.51–14.0) {0.17}	[0.71]	3.75 (0.74–7.15)	9.74 (4.81–11.7)	[0.02]

Data presented as median with range in parenthesis are the ratio of the protein levels of molecules to those of *β*-actin in arbitrary units. Value in square brackets denotes the *p* value of the difference between the normal and tumor tissues of a specific group. Value in curly brackets denotes the *p* value of the difference between the same type of tissue of adenocarcinoma and squamous cell carcinoma.

## Data Availability

The subject's data used to support the findings of this study are restricted by the Institutional Review Board of Chang Gung Memorial Hospital in order to protect patient privacy.
